# Novel land uses shape meta-community structures in neighbouring native forests: Dataset across Uruguay

**DOI:** 10.1016/j.dib.2022.108267

**Published:** 2022-05-16

**Authors:** Ina Säumel, Leonardo R. Ramírez

**Affiliations:** Integrative Research Institute THESys^1^ Transformation of Human-Environment-Systems Humboldt-Universität^2^ zu Berlin, Unter den Linden 6, Berlin 10099, Germany

**Keywords:** Campos region, Clementsian structure, Dispersal limitation, Landscape configuration, Nestedness, Spacial heterogeneity, Species turnover

## Abstract

The presented datasets relate to the research article entitled “Native forest meta-community structures in Uruguay shaped by novel land use types in their surroundings” [Ramírez and Säumel; Ecology and Evolution, 2022]. The datasets include field survey data on woody species presence and absence from 384 plots at 32 permanent monitoring sites of native forests across the Oriental Republic of Uruguay (South America). We compiled different methods from meta-community studies, remote sensing and landscape ecology to explore how woody species communities are influenced by land use change from local to regional scale. We describe the diverse woody species composition in native forests across Uruguay and structure of metacommunities of woody species. Data on woody species diversity inform landscape planning, land-use management, policy and governance and can be used for further meta-analysis with other local, regional or global data sets.

## Specifications Table

**Table:** 

Subject	Ecology
Specific subject area	Ecology of Metacommunities; Remote Sensing; Species composition analysis; Land cover change
Type of data	Table; Image; Chart; Graph; Figure
How the data were acquired	Identification and mapping of woody species during two fieldwork campaigns (from December 2015 to April 2016 and from October 2016 to January 2017) across 32 permanent monitoring sites inside native forest patches of Uruguay;Classification of species occurrence in size/age classes by measurement of dbh, presence based on forest type.Calculation of absolute frequency, relative frequency and cumulative relative frequency of species, elements of Meta-community structure (coherence, turnover and Morisita overlap index) using Matlab [1], distance between sites using ArcGis v.10.3.1 for Desktop [2], Jaccard Index (J) using Past 3.16 [3].Calculation of landscape metrics using Fragstat v.4 [4]: at Landscape scale: number of patches; Landscape shape index, Shannon's evenness index, Aggregation Index; at land use type level: Percentage of the landscape occupied by each land use type; Number of native forest patches within the landscape; Interspersion and juxtaposition index of native forest; Euclidean nearest neighbor distance of native forest; at native forest patch level: Total area of the native forest patch; Perimeter area ratio of native forest patch, Shape index of the native forest patch in a buffer of 3 km from central point of permanent monitoring site
Data format	Raw and analyzed data
Description of data collection	We surveyed woody species diversity at 32 plots of native forests across Uruguay (South America).
Data source location	Oriental Republic of Uruguay (South America)
Data accessibility	Repository name: Edoc Server of the Humboldt Universität zu BerlinData identification number (DOI number): 10.18452/24,171Direct Link: https://doi.org/10.18452/24171
Related research article	L.R. Ramirez, I. Säumel, 2022, Native forest meta-community structures in Uruguay shaped by novel land uses in their surroundings. Ecology and Evolution, 12, e8700. https://doi.org/10.1002/ECE3.8700

## Value of the Data

• The dataset provides relevant information about the main effects of land use change from extensively used grassland to intensively used *Eucalyptus* plantation and agricultural crops on composition of woody species in neighbouring native forests.
• Data on meta-community structure and diversity of woody species are the base to describe the state of the art of the different native forest types and to evaluate how land-use change impacts on these forests.
• Insights from the interactions and influences between meta-community patterns and land-use change inform actors involved in territorial planning, land-use management, policy and governance.
• Data can be used for example for meta-analysis on land-use change impacts on woody species communities with other data sets regarding changes of woody species diversity and land-use change.

## 1 Data Description

The data described in this article show woody species presence and absence, absolute frequency, relative frequency and cumulative relative frequency of species, elements of meta-community structure (coherence, turnover and Morisita overlap index) from 384 plots at 32 permanent monitoring sites of native forests across Uruguay. Native forests cover around 6% of the country's total surface area [5].

Table 1 shows the absolute, relative and cumulative frequency and traits of woody species recorded at 32 permanent monitoring sites across Uruguay. Species are ordered according to absolute frequency (AF) of all species (Total) from higher to lowest values.

**Table 1:** Frequency and traits of woody species recorded at 32 permanent plots across Uruguay (Ramirez and Säumel 2022). AF: absolute frequency, RF: relative frequency (%) and CRF: cumulative relative frequency. Species are ordered according to absolute frequency (AF) of Total from higher to lowest values. * shrub, ^+^ mistletoe and ° liana.

		Total			Adults			Juveniles		
Family	Specie	AF	RF	CRF	AF	RF	CRF	AF	RF	CRF
Sapindaceae	*Allophylus edulis*	31	97	6	28	88	8.7	30	94	6.8
Rhamnaceae	*Scutia buxifolia*	30	94	6	26	81	8.0	22	69	5.0
Myrtaceae	*Blepharocalyx salicifolius*	29	91	6	20	63	6.2	28	88	6.4
Celastraceae	*Maytenus ilicifolia*	21	66	4	1	3	0.3	21	66	4.8
Cannabaceae	*Celtis tala*	19	59	4	10	31	3.1	18	56	4.1
Euphorbiaceae	*Sebastiania brasiliensis*	18	56	3	16	50	5.0	15	47	3.4
Myrtaceae	*Myrcianthes cisplatensis*	18	56	3	16	50	5.0	11	34	2.5
Thymelaeaceae	Daphnopsis racemosa*	18	56	3	1	3	0.3	18	56	4.1
Euphorbiaceae	*Sebastiania commersoniana*	17	53	3	16	50	5.0	16	50	3.7
Sapotaceae	*Pouteria salicifolia*	17	53	3	15	47	4.6	13	41	3.0
Myrtaceae	*Eugenia uniflora*	16	50	3	12	38	3.7	16	50	3.7
Myrtaceae	*Myrrhinium atropurpureum*	14	44	3	8	25	2.5	8	25	1.8
Anacardiaceae	*Lithraea brasiliensis*	12	38	2	11	34	3.4	8	25	1.8
Primulaceae	*Myrsine laetevirens*	11	34	2	9	28	2.8	10	31	2.3
Anacardiaceae	*Schinus longifolia*	11	34	2	8	25	2.5	6	19	1.4
Arecaceae	*Syagrus romanzoffiana*	11	34	2	3	9	0.9	10	31	2.3
Myrtaceae	*Eugenia uruguayensis*	10	31	2	10	31	3.1	9	28	2.1
Verbenaceae	*Citharexylum montevidense*	9	28	2	5	16	1.5	9	28	2.1
Cannabaceae	Celtis iguanaea*	8	25	2				8	25	1.8
Smilacaceae	*Smilax campestris°*	8	25	2				8	25	1.8
Fabaceae	Calliandra tweedii*	7	22	1	1	3	0.3	7	22	1.6
Lythraceae	Heimia salicifolia*	7	22	1				7	22	1.6
Fabaceae	*Erythrina crista-galli*	6	19	1	6	19	1.9	3	9	0.7
Loranthaceae	*Tripodanthus acutifolius^+^*	6	19	1	6	19	1.9			
Lauraceae	*Ocotea acutifolia*	6	19	1	4	13	1.2	5	16	1.1
Oleaceae	*Ligustrum lucidum*	6	19	1	4	13	1.2	4	13	0.9
Myrtaceae	*Myrcianthes pungens*	6	19	1	4	13	1.2	4	13	0.9
Berberidaceae	Berberis laurina*	6	19	1	2	6	0.6	5	16	1.1
Rubiaceae	Guettarda uruguensis*	5	16	1	4	13	1.2	5	16	1.1
Salicaceae	*Salix humboldtiana*	5	16	1	4	13	1.2	3	9	0.7
Sapindaceae	*Cupania vernalis*	5	16	1	3	9	0.9	5	16	1.1
Salicaceae	*Xylosma tweediana*	5	16	1	2	6	0.6	4	13	0.9
Fabaceae	*Gleditsia triacanthos*	4	13	1	3	9	0.9	4	13	0.9
Lamiaceae	*Vitex megapotamica*	4	13	1	3	9	0.9	4	13	0.9
Malvaceae	*Luehea divaricate*	4	13	1	3	9	0.9	3	9	0.7
Lauraceae	*Nectandra megapotamica*	4	13	1	2	6	0.6	4	13	0.9
Verbenaceae	Aloysia gratissima*	4	13	1	1	3	0.3	4	13	0.9
Fabaceae	*Acacia caven*	3	9	1	3	9	0.9	2	6	0.5
Fabaceae	Vachellia caven*	3	9	1	3	9	0.9	2	6	0.5
Quillajaceae	*Quillaja brasiliensis*	3	9	1	2	6	0.6	3	9	0.7
Polygonaceae	*Ruprechtia salicifolia*	3	9	1	2	6	0.6	3	9	0.7
Oleaceae	*Ligustrum sinense*	3	9	1	2	6	0.6	3	9	0.7
Sapindaceae	*Matayba elaeagnoides*	3	9	1	2	6	0.6	2	6	0.5
Lauraceae	*Ocotea puberula*	3	9	1	2	6	0.6	2	6	0.5
Styracaceae	*Styrax leprosus*	3	9	1	2	6	0.6	2	6	0.5
Primulaceae	*Myrsine coriacea*	3	9	1	1	3	0.3	3	9	0.7
Polygonaceae	Ruprechtia laxiflora*	3	9	1	1	3	0.3	3	9	0.7
Rosaceae	*Prunus subcoriacea*	3	9	1	1	3	0.3	2	6	0.5
Fabaceae	*Acacia bonariensis*	3	9	1				3	9	0.7
Rutaceae	*Zanthoxylum rhoifolium*	3	9	1				3	9	0.7
Primulaceae	*Myrsine parvula*	2	6	0.4	2	6	0.6	2	6	0.5
Fabaceae	*Parapiptadenia rigida*	2	6	0.4	2	6	0.6	2	6	0.5
Anacardiaceae	*Schinus mole*	2	6	0.4	2	6	0.6	2	6	0.5
Anacardiaceae	*Lithraea molleoides*	2	6	0.4	2	6	0.6			
Anacardiaceae	*Schinus lentiscifolius*	2	6	0.4	2	6	0.6			
Salicaceae	*Azara uruguayensis*	2	6	0.4	1	3	0.3	2	6	0.5
Rhamnaceae	Colletia paradoxa*	2	6	0.4	1	3	0.3	2	6	0.5
Santalaceae	*Jodina rhombifolia*	2	6	0.4	1	3	0.3	2	6	0.5
Primulaceae	*Myrsine venosa*	2	6	0.4	1	3	0.3	2	6	0.5
Aquifoliaceae	*Ilex paraguariensis*	2	6	0.4	1	3	0.3	1	3	0.2
Myrtaceae	*Myrceugenia glaucescens*	2	6	0.4	1	3	0.3	1	3	0.2
Salicaceae	*Casearia decandra*	2	6	0.4				2	6	0.5
Rubiaceae	Psychotria carthagenensis*	2	6	0.4				2	6	0.5
Fabaceae	*Senna corymbose*	2	6	0.4				2	6	0.5
Symplocaceae	*Symplocos uniflora*	2	6	0.4				2	6	0.5
Myrtaceae	*Calyptranthes concinna*	1	3	0.2	1	3	0.3	1	3	0.2
Salicaceae	*Casearia sylvestris*	1	3	0.2	1	3	0.3	1	3	0.2
Rubiaceae	Cephalanthus glabratus*	1	3	0.2	1	3	0.3	1	3	0.2
Boraginaceae	*Cordia americana*	1	3	0.2	1	3	0.3	1	3	0.2
Asteraceae	*Gochnatia polymorpha*	1	3	0.2	1	3	0.3	1	3	0.2
Moraceae	*Morus alba*	1	3	0.2	1	3	0.3	1	3	0.2
Myrtaceae	*Myrcia palustris*	1	3	0.2	1	3	0.3	1	3	0.2
Phyllanthaceae	Phyllanthus sellowianus*	1	3	0.2	1	3	0.3	1	3	0.2
Fabaceae	*Prosopis affinis*	1	3	0.2	1	3	0.3	1	3	0.2
Myrtaceae	*Acca sellowiana*	1	3	0.2	1	3	0.3			
Fabaceae	*Bauhinia forficate*	1	3	0.2	1	3	0.3			
Arecaceae	*Butia odorata*	1	3	0.2	1	3	0.3			
Cardiopteridaceae	*Citronella gongonha*	1	3	0.2	1	3	0.3			
Escalloniaceae	*Escallonia bifida*	1	3	0.2	1	3	0.3			
Bignoniaceae	*Handroanthus impetiginosus*	1	3	0.2	1	3	0.3			
Phytolaccaceae	*Phytolacca dioica*	1	3	0.2	1	3	0.3			
Euphorbiaceae	*Sapium haematospermum*	1	3	0.2	1	3	0.3			
Anacardiaceae	Schinus engleri*	1	3	0.2	1	3	0.3			
Rutaceae	*Zanthoxylum fagara*	1	3	0.2	1	3	0.3			
Euphorbiaceae	*Actinostemon concolor*	1	3	0.2				1	3	0.2
Arecaceae	*Butia yatay*	1	3	0.2				1	3	0.2
Cannabaceae	Celtis ehrenbergiana*	1	3	0.2				1	3	0.2
Solanaceae	Cestrum parqui*	1	3	0.2				1	3	0.2
Sapotaceae	*Chrysophyllum gonocarpum*	1	3	0.2				1	3	0.2
Cardiopteridaceae	*Citronella paniculate*	1	3	0.2				1	3	0.2
Rhamnaceae	Discaria americana*	1	3	0.2				1	3	0.2
Celastraceae	*Maytenus dasyclados*	1	3	0.2				1	3	0.2
Meliaceae	*Melia azedarach*	1	3	0.2				1	3	0.2
Phytolaccaceae	Phytolacca americana*	1	3	0.2				1	3	0.2
Rutaceae	*Poncirus trifoliata*	1	3	0.2				1	3	0.2
Myrtaceae	Psidium luridum*	1	3	0.2				1	3	0.2
Myrtaceae	Psidium salutare*	1	3	0.2				1	3	0.2
Rosaceae	Pyracantha coccinea*	1	3	0.2				1	3	0.2
Solanaceae	*Solanum mauritianum*	1	3	0.2				1	3	0.2
Solanaceae	*Vassobia breviflora*	1	3	0.2				1	3	0.2
Salicaceae	*Xylosma schroederi*	1	3	0.2				1	3	0.2

In total, we registered 41 families, 77 genera and 101 woody species across native forests of Uruguay (Table 1). The species with higher relative frequency (RF) for adults (A), juveniles (J), and individuals from both age-classes (AJ) were *Allophylus edulis* (Sapindacea, RF: AJ = 97%; *A* = 88%; *J* = 94%), *Scutia buxifolia* (Rhamnaceae, RF: AJ = 94%; *A* = 81%; *J* = 69%) and *Blepharocalyx salicifolius* (Myrtaceae, RF: AJ = 91%; *A* = 63%; *J* = 88%). Of all species, 35 percent occurred only once across all sites.

In total, we registered 41 families, 77 genera and 101 woody species across native forests of Uruguay. The species with higher relative frequency (RF) for adults (A), juveniles (J), and individuals from both age-classes (AJ) were *Allophylus edulis* (Sapindacea, RF: AJ = 97%; *A* = 88%; *J* = 94%), *Scutia buxifolia* (Rhamnaceae, RF: AJ = 94%; *A* = 81%; *J* = 69%) and *Blepharocalyx salicifolius* (Myrtaceae, RF: AJ = 91%; *A* = 63%; *J* = 88%). Of all species, 35 percent occurred only once across all sites.

The total number of woody species per native forest fragment was, for adults, between 4 and 16 (mean = 10.1; SD = 3.4); for juveniles, between 1 and 35 (mean = 13.4; SD = 7.2); and for both age-classes together, between 7 and 37 (mean = 16.3; SD = 6.9). Riverine forests harbor between 7 and 34 (mean = 16.4; SD = 6.6), and hill forests between 10 and 37 species (mean = 17.7; SD = 8.8).

Of all recorded species, 93 percent are native, except seven exotics (i.e. *Gleditsia triacanthos, Ligustrum lucidum, Ligustrum sinense, Morus alba, Melia azedarach, Poncirus trifoliate, Pyracantha coccinea*). More than 70 percent of all species are classified as zoochore (*N* = 72). Nine species are anemochore and eight autochore. Eight species have conservation priority status (i.e. *Ilex paraguariensis, Casearia decandra, Prosopis affinis, Butia odorata, Actinostemon concolor, Maytenus dasyclados, Phytolacca americana, Xylosma* schroederi; [6]; Table 1).

We recorded adults of thirteen native species without any presence of juvenile individuals, among them *Butia odorata*, which is categorized as high priority for conservation (Table 1). All occur with low frequency, except the hemiparasitic mistletoe *Tripodanthus acutifolius*.

Of the species, 26 were recorded only in the regeneration layer but not among adults. All are native to the region, except the South-East Asian *Melia azedarach,* the Chinese *Poncirus trifoliata* and the European *Pyracantha coccinea* (Table 1). Most frequent species are the climbing *Celtis iguanaea, Smilax campestris* and the shrubby *Heimia salicifolia.* Five of the native species that only occurred in the regeneration layer have conservation priority (i.e. *Casearia decandra, Actinostemon concolor, Maytenus dasyclados, Phytolacca americana* and *Xylosma schroederi*). In addition, we recorded 27 species only at one site as adults (i.e. *Acca sellowiana, Bauhinia forficata, Butia odorata, Citronella gongonha, Escallonia bífida, Handroanthus impetiginosus, Phytolacca dioica, Sapium haematospermum, Schinus engleri, Zanthoxylum fagara*), 17 species only at one site in the regeneration layer (i.e. *Actinostemon concolor, Butia yatay, Celtis ehrenbergiana, Cestrum parqui, Chrysophyllum gonocarpum, Citronella paniculata, Discaria americana, Maytenus dasyclados, Melia azedarach, Phytolacca americana, Poncirus trifoliata, Psidium luridum, Psidium salutare, Pyracantha coccinea, Solanum mauritianum, Vassobia breviflora, Xylosma schroederi*), and 9 species only at one site but as adults and juvenile (*Calyptranthes concinna, Casearia sylvestris, Cephalanthus glabratus, Cordia americana, Gochnatia polymorpha, Morus alba, Myrcia palustris, Phyllanthus sellowianus, Prosopis affinis*; Table 1).

**Fig. 1:**
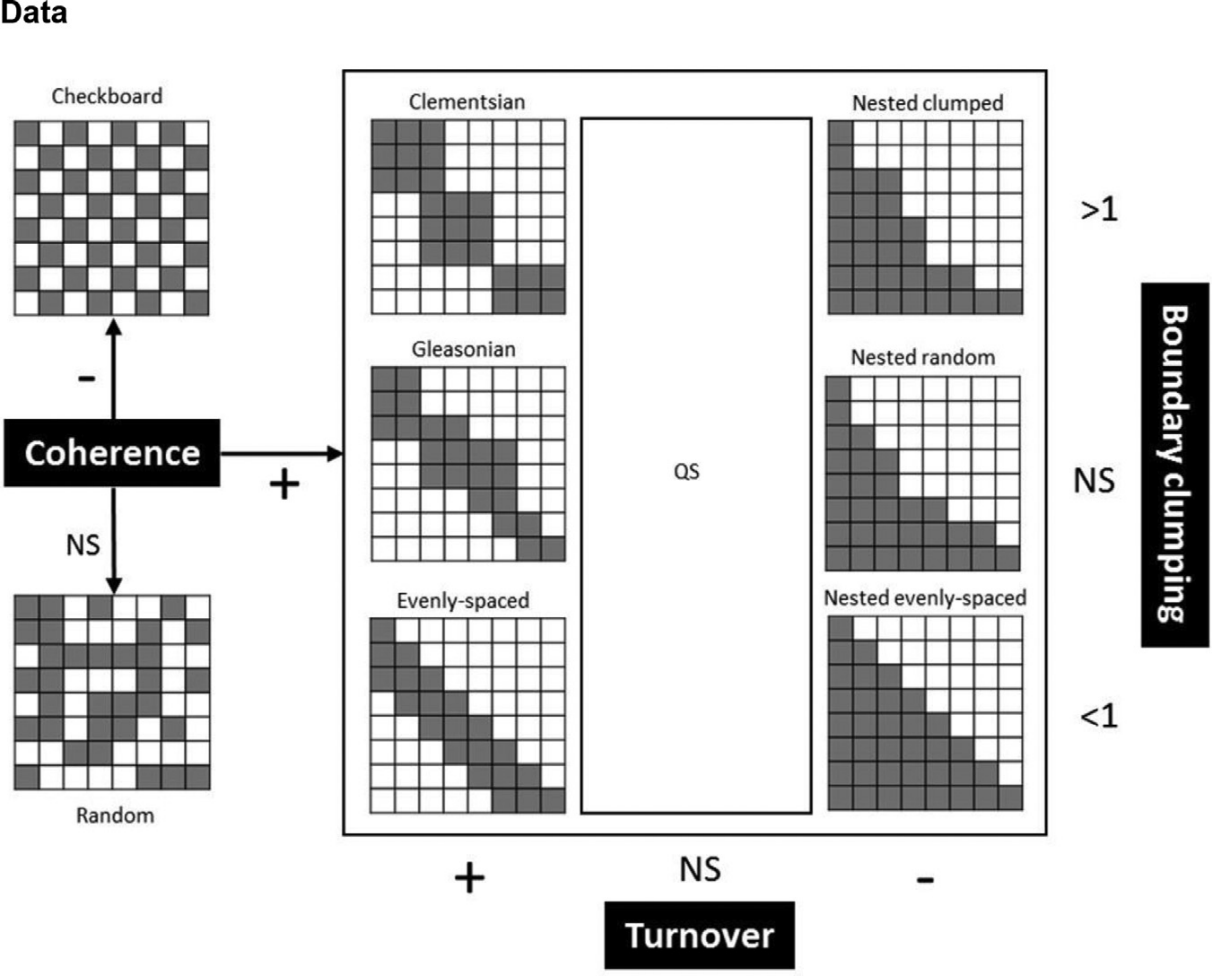
Scheme of idealized pattern of species distribution (checkboard, random, clementsian, gleasonian, evenly-spaced, nested clumped, nested random, nested evenly-spaced and QS or quasi-structures) where columns represent sites and rows represent species, gray square mean specie presence and white mean specie absence (based on [7,8]). Steps to determine the pattern of species distribution are (1) observation of species coherence, (2) evaluation of species turnover and (3) analysis of boundary clumping using Morisita overlap index. NS = non-significant, “+” = significantly positive, “-” = significantly negative.

Fig. 1 shows a scheme of idealized pattern of species distribution (checkboard, random, clementsian, gleasonian, evenly-spaced, nested clumped, nested random, nested evenly-spaced and QS or quasi-structures) where columns represent sites and rows represent species, gray square mean specie presence and white mean specie absence (based on [7,8]). Species distribution among sites can follow a discrete or Clementsian pattern [9], a continuous or Glesonian pattern [10], a random pattern [11], a checkboard pattern [12], evenly-spaced patterns [13], nested subset [14], and mixed pattern between nested-random or nested evenly-spaced [8]. Steps to determine the pattern of species distribution are shown: (1) observation of species coherence, (2) evaluation of species turnover and (3) analysis of boundary clumping using Morisita overlap index. NS = non-significant, “+” = significantly positive, “-” = significantly negative.

**Fig. 2:**
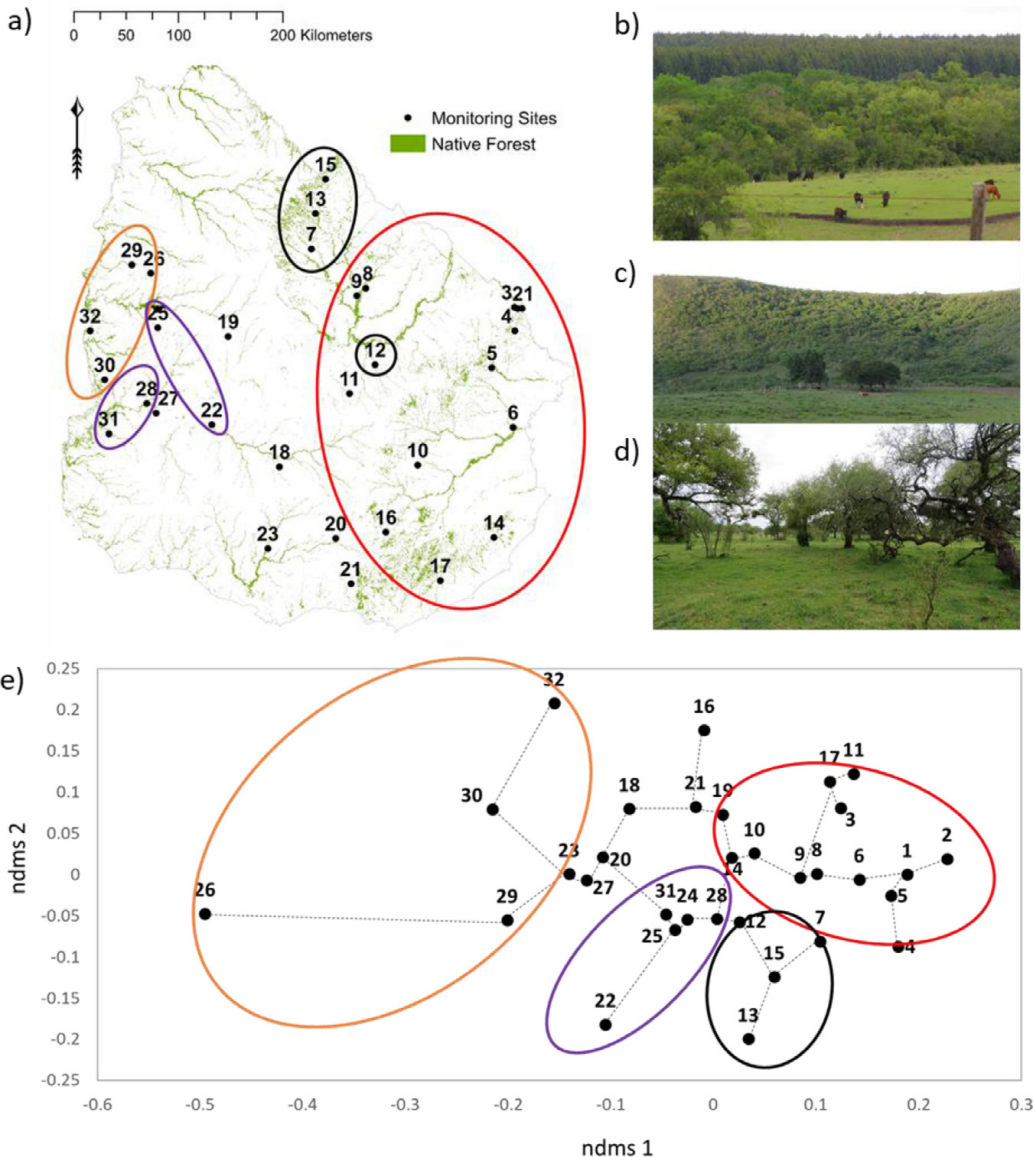
(a) Distribution of the 32 permanent monitoring sites of native forests across Uruguay, South America, (b) Riverine forests surrounded by grassland and timber plantation, (c) hill forest, (d) park forest and (e) NDMS (non-metric multi-dimensional scaling) using Jaccard distance between native forest. Numbers in (a) and (e) indicate the ID of permanent monitoring sites, ellipses in (a) and (e) indicate the visual grouping based on minimum spanning tree algorithm.

Fig. 2 shows the distribution of native forests and the 32 permanent monitoring sites in different native forests across Uruguay, South America. photographs show a riverine forest surrounded by grassland and timber plantation (b), a hill forest (c) and an example for park forests (d). Moreover, Fig. 2e shows the non-metric multi-dimensional scaling (NDMS) using Jaccard distance between native forest.

**Fig. 3:**
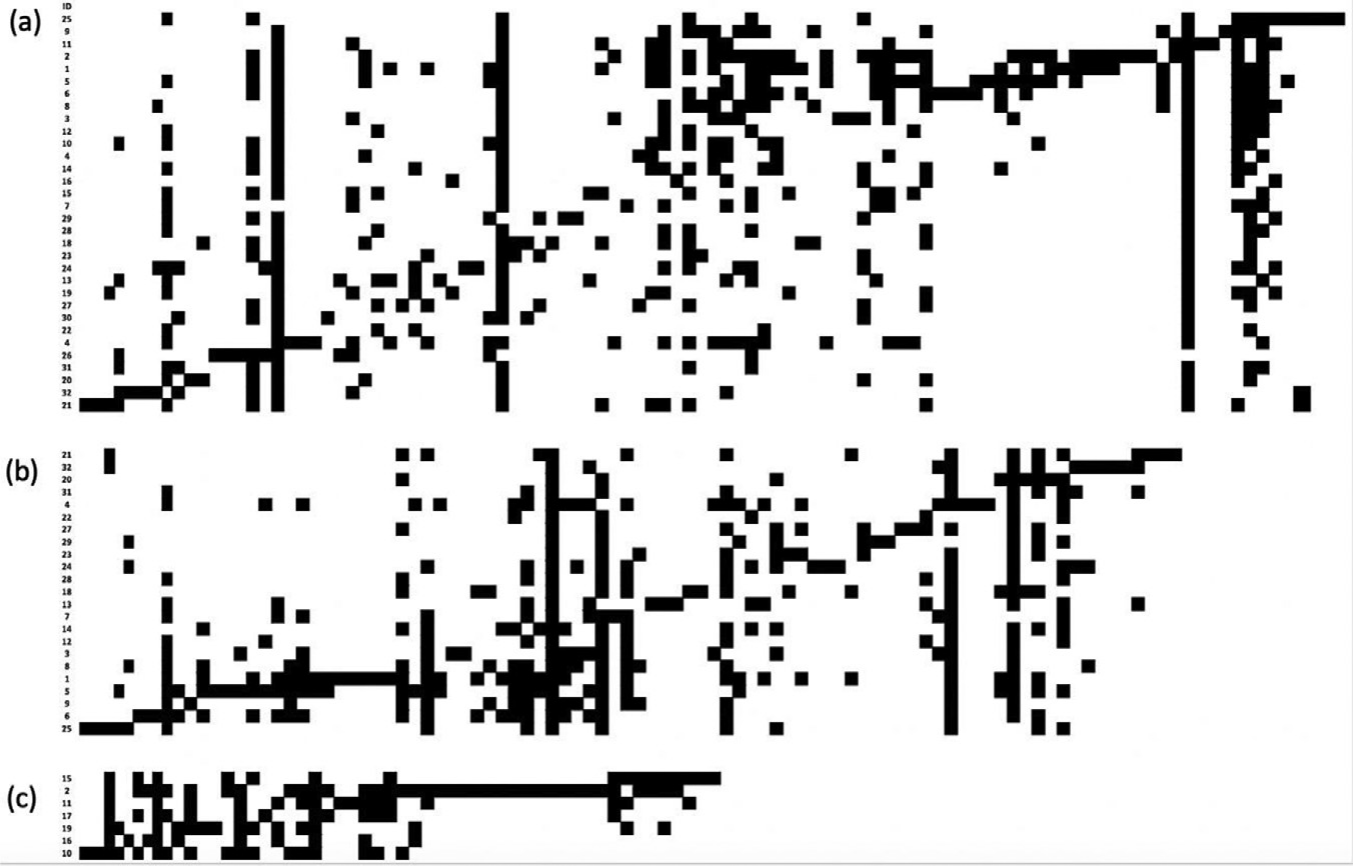
Matrix ordination by reciprocal averaging of both age classes together (juveniles and adults) for all native fragments under study (Ramirez and Säumel 2022; (a), riverine forest (b) and hill forest (c). Rows = monitoring sites, columns = species, black cell = presence and white cells = absence. Numbers indicate the ID of permanent monitoring sites (see Fig. 2a).

**Fig. 4:**
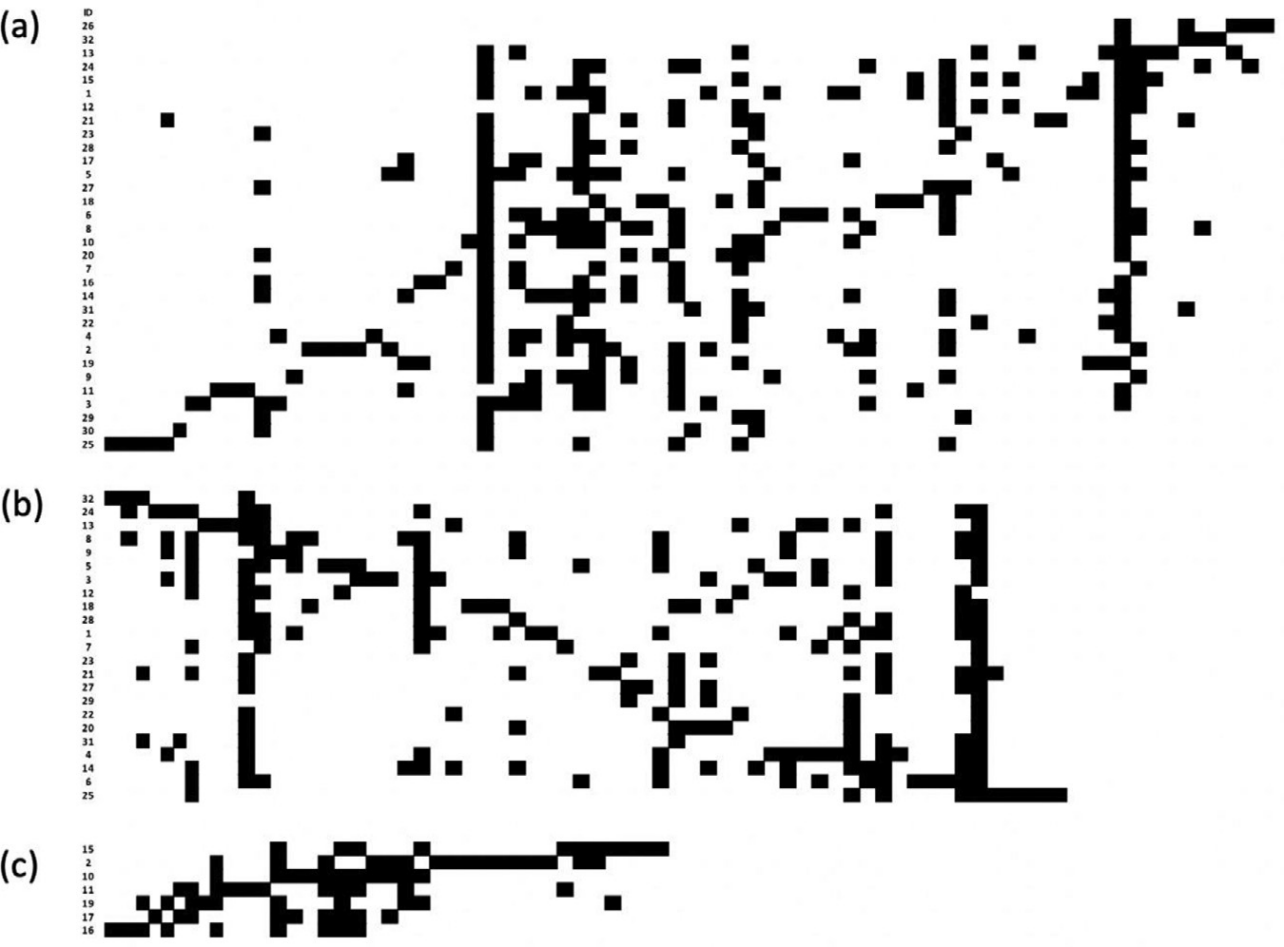
Matrix ordination by reciprocal averaging of adults for all native fragments under study (a), riverine forest (b) and hill forest (c). Rows = monitoring sites, columns = species, black cell = presence and white cells = absence. Numbers indicate the ID of permanent monitoring sites (see Fig. 2a).

**Fig. 5:**
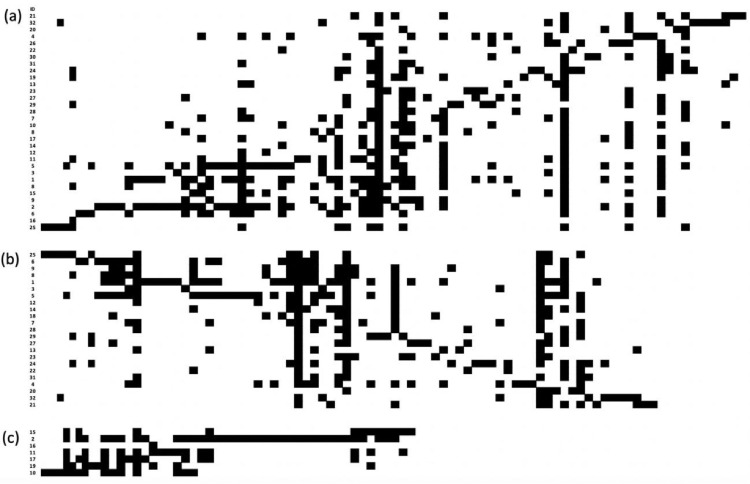
Matrix ordination by reciprocal averaging of juveniles for all native fragments under study (a), riverine forest (b) and hill forest (c). Rows = monitoring sites, columns = species, black cell = presence and white cells = absence. Numbers indicate the ID of permanent monitoring sites (see Fig. 2a).

**Fig. 6:**
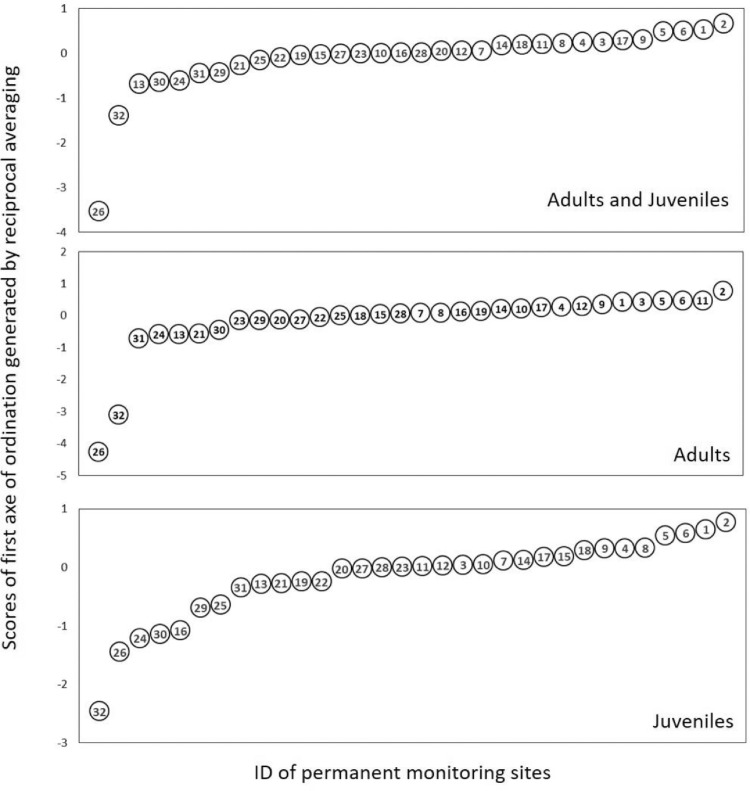
Scores of first axis of ordination generated by reciprocal averaging for adults, juvenils and for all woody species versus ID of the monitoring sites. For ID of monitoring sites (see Fig. 2a).

In Fig. 3, Fig. 4, Fig. 5, Fig. 6 we show the matrix ordination by reciprocal averaging of the different frequencies of juveniles, adults and of both age classes together (juveniles and adults) for riverine (23), hill (7) forests and for all (32) native fragments under study (rows) and species recorded (columns). Black cells indicate presence and white cells absence.

Table 2 shows the linear distance matrix between permanent plots (or sites) in kilometer (below of diagonal) and Morisita index (above of diagonal). ID number represents each forest fragments. The matrix was ordered according linear distance between 1 and 32 sites (see column 1) and Fig. 2.

Table 3 shows the scores of first axis of ordination generated by reciprocal averaging.

Table 4 shows the department and sites of each permanent plots (ID) across Uruguay and overview on metadata of landscape metric per site. ID = code native forest fragments (Fig. 1). Means are given at landscape scale for number of patches (NP): number of patches (NP); Landscape shape index (LSI), Shannon's evenness index (SHEI), Aggregation Index (AI) and at land use type level: Percentage of the landscape (P) occupied by each land use type (NF: native forest; GL: Grassland; TF: Timber forest; C: crops); Number of native forest patches within the landscape (NNF); Interspersion and juxtaposition index of native forest (IJI); Euclidean nearest neighbor distance of native forest (ENN) in a buffer of 3 km from central point of permanent monitoring site.

Raw Data are uploaded in the Open Access repository of the Humboldt Universität zu Berlin (https://edoc.hu-berlin.de) as Säumel, I. and Ramírez, L. 2021: Land use change impacts on meta-community structures in Uruguayan native forests.

## 2 Experimental Design, Materials and Methods

Woody diversity datasets were obtained from two fieldwork campaigns (from December 2015 to April 2016 and from October 2016 to January 2017) across 32 permanent monitoring sites inside native forest patches of Uruguay (Fig. 2).

In general, we used a stratified randomized design. In a first step, we randomly selected monitoring sites across the country and then stratified by different land use types (i.e. native forests, grassland, timber plantation, crops). Second, we asked the potential land owners for their willingness to establish long term monitoring sites. In total we established 32 long-term monitoring plots (100 × 100 m) in different native forests fragments across Uruguay (23 sites with riverine and seven hill forests; Fig. 2).

In the vegetations periods 2015/2016 and 2016/2017, we recorded all woody species in two size-classes based on diameter at breast height (dbh). We take the size-classes as a non-invasive proxy measure for tree age to differentiate in adults (dbh ≥ 5 cm) recorded in 3 plots of 10 × 20 m and juveniles (dbh < 5 cm) recorded in 9 plots of 3 × 3 m. Thus, juvenile plots were nested within adults. The woody species in the local forests comprise also multi-stem species, that there are 64 species categorized as trees, 21 as shrubs and 32 that form the growth habit as shrubs or trees depending on site conditions (*e.g. Blepharocalyx salicifolius, Eugenia uniflora or Maytenus ilicifolia*). Classification in shrubs, trees and those species that can have both growth habits are indicated in Table 1. All names of species identified were updated using the online database from [15].

The meta-community structure was described by different elements of meta-community structure (EMS; Fig. 1; [7,8]): coherence (i.e. number of interruptions in species distribution across the sites), species turnover (i.e. number of species replacements between two sites) and boundary clumping (i.e. boundaries in species composition across two or more sites based on Morisita overlap index). When coherence is negative or not significant, the meta-community follows a checkboard or random pattern respectively. When coherence is statistically significant and positive (*p* < 0.05; less embedded absences than expected by chance), the meta-community is classified into six basic structures evaluating turnover and boundary clumping. When turnover is statistically significant and negative (*p* < 0.05; less replacements than expected by chance), the meta-community can follow some nested pattern (i.e. evenly-spaced, clumped or random). When turnover is statistically significant and positive (*p* < 0.05; with more replacements than expected by chance), the meta-community is classified as a Clementsian, Gleasonian or evenly-spaced pattern (Fig. 1). The Morisita index (MI) needs to be evaluated to determine boundary clumping between different woody communities (if MI > 1, a Clementsian structure and if MI < 1, an evenly spaced structure; [7,8]). The EMS were calculated with Matlab [1], using a script developed by Presley and Higgins [16].

We determined the elements of meta-community structure (EMS) for matrix of adult individuals, juvenile individuals of the regenerating layer and total species (sum of adult and juvenile woody species). The models for matrix ordination were set by reciprocal averaging (Fig. 3–11; [17]), the null model with fixed species richness per site and equiprobable species occurrence (random 0). The models ran with 1000 iteration and extraction of the scores from first axes of ordination based on reciprocal averaging.

For Table 2 we created a matrix distance-species distribution pattern to explore whether geographic distance influenced the species composition between sites. The distance between sites was calculated using ArcGis v.10.3.1 for Desktop [2] and boundary clumbing between different communities was calculated based on Morisita index (MI; see Fig. 1) using Past 3.16 [3]. The matrix distance- species distribution pattern was calculated to both age-classes together.

For Fig. 2 the Jaccard similarity coefficient matrix was subjected to non-metric multidimensional scaling ordination (NMDS) to assess species assemblage among different native forest types. We used a matrix distance-similarity to explore whether geographic distance influenced the similarity of species composition between sites. The distance between sites was calculated using ArcGis v.10.3.1 for Desktop [2] and composition (di)similarity was calculated based on Jaccard Index (J) using Past 3.16 [3]. The matrix distance-similarity was calculated to both age-classes together.

**Table 2:** Linear distance matrix between permanent plots (or sites) in kilometer (below of diagonal) and Morisita index (above of diagonal). ID number represents each forest fragments (Fig. 2a). The matrix was ordered according linear distance between 1 and 32 sites (see column 1).

ID	1	2	3	4	5	6	7	8	9	10	11	12	13	14	15	16	17	18	19	20	21	22	23	24	25	26	27	28	29	30	31	32
1		.40	.29	.15	.53	.32	.45	.41	.31	.44	.18	.86	.77	.41	.83	.31	.38	.41	.05	.11	.10	.03	.34	.50	.04	.05	.21	.55	.01	.01	.02	.01
2	4		.83	.36	.39	.14	.83	.85	.83	.57	.55	.51	.14	.71	.15	.13	.06	.75	.11	.00	.01	.06	.01	.23	.01	.00	.00	.74	.00	.00	.01	.00
3	7	3		.35	.24	.06	.94	.94	.96	.58	.62	.41	.00	.74	.01	.08	.08	.80	.16	.01	.01	.00	.04	.22	.01	.00	.06	.76	.01	.01	.00	.00
4	23	21	23		.12	.15	.46	.36	.33	.49	.24	.26	.05	.46	.15	.21	.18	.32	.26	.77	.79	.55	.06	.10	.04	.01	.04	.53	.06	.01	.81	.00
5	64	62	62	42		.56	.31	.40	.32	.38	.27	.48	.33	.36	.42	.14	.17	.29	.17	.05	.04	.48	.16	.39	.01	.03	.12	.36	.00	.00	.01	.00
6	114	114	115	92	60		.06	.26	.09	.60	.15	.10	.03	.47	.09	.61	.76	.24	.05	.16	.17	.40	.47	.29	.07	.07	.50	.25	.01	.01	.04	.01
7	151	147	144	138	112	145		.92	.93	.63	.64	.56	.19	.76	.20	.13	.07	.77	.28	.13	.14	.09	.02	.34	.03	.00	.00	.79	.01	.00	.14	.00
8	151	147	143	148	143	194	74		.94	.73	.59	.45	.04	.85	.06	.18	.25	.87	.12	.07	.06	.13	.19	.26	.02	.03	.17	.84	.00	.00	.01	.01
9	159	154	151	155	146	196	68	11		.57	.68	.44	.04	.77	.06	.04	.08	.78	.20	.00	.01	.03	.01	.27	.01	.00	.14	.76	.00	.00	.01	.00
10	180	178	177	159	117	98	104	176	172		.42	.30	.03	.90	.08	.70	.65	.64	.18	.31	.38	.25	.38	.37	.11	.04	.29	.65	.05	.06	.18	.01
11	184	180	178	169	138	160	37	102	94	95		.31	.00	.67	.02	.16	.19	.52	.71	.01	.01	.00	.04	.68	.03	.00	.21	.50	.00	.00	.01	.00
12	210	206	202	210	207	258	127	64	62	231	143		.73	.37	.79	.05	.07	.49	.15	.11	.10	.07	.09	.46	.02	.02	.07	.67	.01	.00	.15	.00
13	218	214	211	221	224	279	156	86	88	260	176	34		.02	.88	.03	.02	.00	.02	.04	.04	.04	.01	.30	.01	.01	.00	.18	.00	.00	.05	.00
14	221	220	221	199	162	107	201	268	266	101	195	327	354		.07	.47	.54	.75	.27	.21	.24	.15	.28	.42	.07	.03	.37	.76	.05	.04	.13	.01
15	225	222	219	232	241	298	184	111	116	288	207	68	34	379		.06	.08	.04	.05	.15	.15	.13	.06	.35	.01	.01	.08	.25	.01	.00	.16	.00
16	251	249	248	229	187	158	161	234	228	71	137	280	312	104	343		.76	.15	.20	.17	.25	.03	.39	.38	.14	.04	.25	.13	.10	.08	.04	.01
17	272	271	271	250	210	163	216	289	284	113	199	341	371	66	400	70		.29	.04	.22	.23	.03	.63	.33	.09	.09	.63	.30	.02	.04	.05	.02
18	277	274	272	260	224	227	134	190	180	132	97	211	245	216	279	120	189		.10	.17	.06	.01	.35	.28	.02	.06	.23	.91	.00	.01	.05	.01
19	283	278	275	274	254	286	143	140	129	219	128	116	144	319	177	241	310	134		.22	.23	.16	.03	.74	.05	.00	.02	.15	.02	.00	.25	.00
20	283	281	280	262	221	200	170	241	233	105	139	278	312	151	344	48	108	87	219		.80	.51	.26	.10	.03	.03	.23	.31	.17	.15	.76	.01
21	310	308	308	288	247	216	211	283	276	130	182	323	356	144	388	60	85	131	264	46		.50	.16	.09	.06	.03	.15	.26	.10	.10	.74	.03
22	317	313	311	304	273	288	166	197	186	201	135	193	225	291	259	196	265	76	86	161	202		.02	.01	.01	.00	.01	.16	.04	.00	.58	.00
23	335	332	330	315	275	262	204	266	257	164	168	290	324	217	358	114	168	79	207	66	86	130		.29	.03	.12	.60	.35	.38	.23	.03	.02
24	349	345	342	343	325	359	215	200	191	290	201	158	177	389	203	306	375	191	73	278	322	122	252		.06	.07	.20	.34	.01	.01	.02	.19
25	349	345	342	342	322	353	211	203	193	281	194	165	186	379	215	293	363	177	68	264	307	106	236	18		.00	.02	.02	.01	.01	.03	.00
26	357	353	350	353	339	377	232	206	198	315	222	155	168	415	190	335	404	223	96	310	354	156	287	35	53		.07	.06	.00	.00	.00	.01
27	364	360	358	352	324	342	215	234	222	255	186	216	244	345	277	248	316	129	101	210	248	54	168	100	82	134		.28	.36	.36	.04	.01
28	371	366	364	359	332	351	222	237	226	266	194	216	243	356	275	260	328	141	101	222	261	65	181	91	73	125	13		.02	.00	.28	.01
29	376	372	368	372	358	397	252	225	217	334	242	172	182	434	203	353	423	239	115	327	371	171	301	49	65	20	144	134		.71	.06	.00
30	405	401	398	395	371	394	259	265	254	311	235	234	257	402	286	306	375	187	125	268	306	111	225	84	71	111	59	46	113		.01	.00
31	413	409	407	401	372	387	263	282	271	297	233	262	289	382	320	281	347	166	147	239	273	99	188	128	112	159	49	46	163	52		.00
32	414	410	407	406	386	415	275	267	257	339	255	226	243	434	268	342	412	223	132	308	348	147	269	68	65	80	101	88	75	49	100

**Table 3:** The scores of first axis of ordination generated by reciprocal averaging for adults, juvenils and all age classes together. Numbers indicate the ID of permanent monitoring sites (see Fig. 2a) and the approx. latitude and longitude. We cannot publish the exact location as we agreed to protect owner privacy.

ID	Latitude	Longitude	Adults and Juveniles	Adults	Juveniles
1	−32	−54	0.527167	0.414966	0.647240
2	−32	−54	0.660633	0.764692	0.775401
3	−32	−54	0.245467	0.431703	0.040260
4	−32	−54	0.239254	0.267534	0.325141
5	−33	−54	0.475965	0.458633	0.541064
6	−33	−54	0.491153	0.464190	0.582062
7	−33	−55	0.049349	0.082344	0.108956
8	−32	−55	0.222261	0.083222	0.329336
9	−32	−55	0.306056	0.354837	0.322345
10	−34	−55	−0.002515	0.205924	0.047635
11	−33	−55	0.200543	0.469457	0.015936
12	−32	−56	0.043390	0.295778	0.030319
13	−31	−56	−0.687450	−0.585130	−0.284849
14	−34	−54	0.177360	0.204617	0.119347
15	−31	−56	−0.037545	0.036918	0.185851
16	−34	−55	0.002131	0.118208	−1.083550
17	−35	−54	0.280042	0.254687	0.181019
18	−34	−56	0.193801	0.003753	0.290895
19	−32	−57	−0.052246	0.131023	−0.246512
20	−34	−56	0.043060	−0.137313	−0.025985
21	−35	−55	−0.275978	−0.571120	−0.270884
22	−33	−57	−0.098342	−0.050924	−0.244235
23	−34	−56	−0.007416	−0.150409	0.005323
24	−32	−57	−0.619125	−0.591702	−1.211210
25	−32	−57	−0.156738	0.002066	−0.642816
26	−32	−58	−3.537520	−4.259300	−1.449460
27	−33	−57	−0.022680	−0.132921	−0.019935
28	−33	−58	0.008329	0.060685	0.000694
29	−32	−58	−0.431746	−0.139724	−0.693187
30	−33	−58	−0.638588	−0.444915	−1.141800
31	−33	−58	−0.450609	−0.710617	−0.348537
32	−32	−58	−1.392330	−3.112280	−2.462570

**Table 4:** Department and site each permanent plots (ID) across Uruguay (Fig. 2a) and overview on metadata of landscape metric per site. ID = code native forest fragments (Fig. 1). Means are given at landscape scale for number of patches (NP): number of patches (NP); Landscape shape index (LSI), Shannon's evenness index (SHEI), Aggregation Index (AI) and at land use type level: Percentage of the landscape (P) occupied by each land use type (NF: native forest; GL: Grassland; TF: Timber forest; C: crops); Number of native forest patches within the landscape (N_NF_); Interspersion and juxtaposition index of native forest (IJI); Euclidean nearest neighbor distance of native forest (ENN) in a buffer of 3 km from central point of permanent monitoring site.

Department	ID	NP	LSI	SHEI	AI	P_NF_	P_GL_	P_TF_	P_C_	P_TP&C_	N_NF_	IJI	ENN
Cerro Largo	1	133	7.7	0.394	92.6	15.6	82.0	0.0	0.0	0.5	100	33.2	104.7
Cerro Largo	2	96	3.5	0.127	97.3	2.7	96.0	1.0	1.0	1.3	62	20.5	211.9
Cerro Largo	3	143	6.0	0.351	94.6	5.6	83.9	1.3	1.3	1.4	81	42.9	152.2
Cerro Largo	4	128	7.6	0.427	92.8	15.7	8.4	3.9	3.9	3.9	76	41.0	131.3
Treinta y Tres	5	100	7.4	0.583	93.1	9.9	61.6	27.5	27.5	27.9	27	58.2	200.2
Treinta y Tres	6	236	14.4	0.827	85.3	36.8	21.5	0.0	0.0	39.6	32	53.1	87.3
Cerro Largo	7	177	8.6	0.475	91.8	11.4	76.8	9.9	9.9	1.7	89	40.1	112.1
Tacuarembó	8	175	8.1	0.688	92.4	45.7	4.5	0.4	0.4	7.8	59	60.8	125.6
Tacuarembó	9	147	8.7	0.698	91.8	46.8	36.1	0.1	0.1	13.1	30	59.7	184.4
Lavalleja	10	161	9.7	0.615	90.5	13.5	67.7	18.8	18.8	18.8	52	25.8	150.8
Durazno	11	250	9.5	0.477	90.6	9.2	79.5	2.0	2.0	11.3	158	56.3	93.9
Tacuarembo	12	158	8.4	0.493	91.9	11.5	72.0	16.4	16.4	16.3	92	40.7	139.3
Tacuarembo	13	115	6.6	0.721	94.1	13.8	49.3	36.8	36.8	36.9	43	55.9	189.7
Rocha	14	117	7.8	0.512	92.7	7.4	79.6	11.5	11.5	13.6	59	9.0	209.0
Rivera	15	148	9.4	0.708	91.0	15.0	28.5	51.9	51.9	56.9	51	57.1	162.0
Lavalleja	16	253	13.6	0.848	86.1	22.3	61.3	16.4	16.4	16.4	153	69.3	101.8
Rocha	17	247	14.6	0.658	84.9	7.3	63.9	26.6	26.6	28.8	67	49.8	148.3
Florida	18	101	8.1	0.523	92.3	8.9	67.2	0.2	0.2	24.4	19	41.9	285.0
Rio Negro	19	329	15.2	0.652	84.4	12.8	54.4	31.2	31.2	33.3	172	50.7	85.9
Florida	20	93	7.2	0.580	93.3	5.8	66.5	0.0	0.0	27.6	29	33.5	236.8
Lavalleja	21	397	13.5	0.684	86.3	6.7	25.3	12.0	12.0	67.9	65	71.9	193.9
Flores	22	131	10.0	0.701	90.3	14.9	63.1	11.3	11.3	18.4	67	61.1	123.6
Florida	23	258	14.0	0.924	85.8	18.5	32.5	0.0	0.0	38.8	83	74.9	121.7
Paysandu	24	119	8.0	0.589	92.5	56.6	34.5	0.8	0.8	7.6	43	45.2	103.1
Paysandú	25	203	13.9	0.673	85.7	8.5	52.6	38.5	38.5	38.8	76	54.5	89.5
Paysandu	26	306	13.8	0.712	85.8	7.5	35.7	54.3	54.3	56.9	173	66.7	111.4
Soriano	27	146	8.7	0.589	91.7	0.7	23.8	18.6	18.6	19.2	30	54.9	289.2
Rio Negro	28	154	11.7	0.837	88.5	26.6	46.3	12.7	12.7	21.1	32	56.7	134.5
Paysandú	29	232	11.2	0.883	88.9	14.4	4.9	36.1	36.1	44.7	113	67.1	95.8
Rio Negro	30	189	11.1	0.827	89.0	13.7	56.9	18.7	18.7	29.4	80	33.6	106.4
Soriano	31	182	10.8	0.784	89.4	9.4	61.9	13.4	13.4	29.5	32	50.7	236.7
Paysandú	32	203	12.1	0.572	87.8	1.1	19.5	0.0	0.0	67.9	34	69.6	200.0

We classified land use from Landsat 8 OLI satellite image for the year 2017 [17] in a buffer zone of 3 km from central point of each permanent plot, processing atmospheric and geometric correction by Landsat image using Matlab [1]. We combined two techniques of classification: we first used supervised classification using ground control points collected in field across different land uses to capture signature spectral of each land use type, then used tree classification technics based on signature spectral of each land use type with Envy v.5.3 [18]. The land use maps were set to six land use types (i.e. native forest, grassland, timber plantation, agriculture, water body and urban areas). Due to the small area covered by water bodies and urban areas, these land uses were not considered in the analysis. For details see Ramírez and Säumel (2022).

At landscape level, we calculated total number of patches (total number of patches in the landscape without considering the identity of the land use type), landscape shape index (standardized measure of total edge that adjusts for the size of the landscape, where index increases without limit as landscape shape becomes more irregular and/or as the length of edge within the landscape increases), Shannon's evenness index (distribution of area among patch types where larger values mean higher landscape diversity), and Aggregation Index (frequency with which different pairs of patch types appear side-by-side on the map). At class level, we calculated percentage of the landscape occupied by each land use type and the metrics of native forests without considering other land use types within of each buffer. We thus determined number of native forest patches (number of native forest patches within the landscape), interspersion and juxtaposition index (measure at class level based on patch adjacencies), Euclidean nearest neighbor distance (mean of the shortest straight-line distance between all native forest patches by landscape). Finally, at patch level, we only considered the native forest fragment where the permanent plots were established, thus calculating total area, perimeter area ratio as a measure of shape complexity, and shape index of patch as a measure of compactness. All spatial metrics were calculated for the 3 km buffer using Fragstat v.4 [4].

The landscape shape index (LSI) by land-use type is given by:

(1) $$\text{LSI}=\;\frac{0.25\sum_{k=1}^{m}e_{\text{ik}}^{*}}{\sqrt{A}}$$

Where $e_{ik}^{*}$ is the total length of edges in the landscape between patches types of land-use type i and k, A is the total area of landscape and 0.25 is the factor of adjustment for raster format.

The Shannon's evenness index (SHEI) is given by:

(2) $$\text{SHEI}=\frac{-\sum_{n=1}^{m}\left(P_{i}*\text{ln}P_{i}\right)}{\text{ln}\;m}$$

Where $P_{i}$ is the proportion of the landscape occupied by patch type belonging to land use tipe i and m number of patches of land use type in the landscape

The aggregation index (AI) expressed in percentage is given by:

(3) $$\text{AI}=\left\lfloor \frac{g_{\text{ii}}}{\max\to g_{\text{ii}}}\right\rfloor \left(100\right)\;$$

Where $g_{\text{ii}}$ is the number of joins between pixel of patches belonging to land-use type i, $\max\to g_{\text{ii}}$ is the maximum number of joins between pixels of the same land-use type i.

The percentage of landscape occupied by each land-use type is given by:

(4) $$P_{i}=\;\frac{\sum_{j=1}^{n}a_{\text{ij}}}{A}\left(100\right)$$

Where $P_{i}$ is the proportion of the landscape occupied by patches belonging to land-use i, $a_{\text{ij}}\;$is the area of patchij, A is the total area of the landscape.

The Interspersion and juxtaposition index (IJI) of native forest is given by:

(5) $$\text{IJI}=\;\frac{-\sum_{k=1}^{m}\left[\left(\frac{e_{\text{ik}}}{\sum_{k=1}^{m}e_{\text{ik}}}\right)\text{ln}\left(\frac{e_{\text{ik}}}{\sum_{k=1}^{m}e_{\text{ik}}}\right)\right]}{\ln\left(m-1\right)}\left(100\right)$$

Where $e_{\text{ik}}$is the total edge lenght (m) in the landscape between native forest patches i and k, m is the number of native forest parches present in the landscape

The Mean Euclidean nearest neighbor distance (ENN_MN) is given by:

(6) $$\text{ENN}\_\text{MN}=\;\frac{\sum h_{\text{ij}}}{n}$$

Where $h_{\text{ij}}$is the distance (m) from ij to nearest neighboring patch of same type and n is the number of nearest neighbouring distance The ENN_MN is based on patch edge-to-edge distance and computed from cell to cell center of patches

The Perimeter area ratio (PARA) is given by:

(7) $$\text{PARA}=\;\frac{P_{\text{ij}}}{a_{\text{ij}}}$$

Where Pij is the perimeter (m) of patch ij and aij is the area (m^2^) of patch ij.

The Shape index of patch (SHAPE) is given by:

(8) $$SHAPE=\;\frac{0.25\;P_{ij}}{\sqrt{a_{ij}}}$$

Where Pij is the perimeter (m) of the native forest patch ij and aij is the area (m^2^) of the native forest patch ij.

## Ethics Statements

The authors comply with the ethical guidelines of the journal. Humans, animals or data from social media are not involved in this research.

## CRediT authorship contribution statement

**Ina Säumel:** Conceptualization, Methodology, Investigation, Visualization, Writing – original draft, Validation. **Leonardo R. Ramírez:** Conceptualization, Methodology, Data curation, Writing – original draft, Visualization, Investigation, Validation.

## Declaration of Competing Interest

The authors declare that they have no known competing financial interests or personal relationships that could have appeared to influence the work reported in this paper.

## Data Availability

Land use change impacts on metacommunity structures in Uruguayan native forests (Original data) (Edoc HU Berlin).
